# geomeTriD: a Bioconductor package for interactive and integrative visualization of 3D structural model with multi-omics data

**DOI:** 10.1093/bioadv/vbaf299

**Published:** 2025-11-23

**Authors:** Jianhong Ou, Kenneth D Poss

**Affiliations:** Morgridge Institute for Research, Madison, WI 53715, United States; Morgridge Institute for Research, Madison, WI 53715, United States; Department of Cell and Regenerative Biology, School of Medicine and Public Health, University of Wisconsin-Madison, Madison, WI 53715, United States

## Abstract

**Motivation:**

The three-dimensional organization of the genome plays a critical role in regulating gene expression by shaping the spatial and temporal interactions between regulatory elements. High-throughput chromosome conformation capture (Hi-C) technologies, along with immunoprecipitation- or chromatin accessibility-based chromatin architecture mapping methods, enable the measurement of chromatin dynamics at both bulk and single-cell levels. However, effectively exploring and comparing chromatin structures remains challenging, particularly when integrating multiple layers of genomic annotation or comparing structural dynamics across conditions. While several tools support interactive 3D genome visualization, few provide a flexible, R-integrated framework that supports custom annotations, side-by-side comparison of multiple stages or conditions, and deployment in Shiny applications.

**Results:**

To address this need, we have developed geomeTriD, an R/Bioconductor package that enables interactive visualization of chromatin structures using three.js, supports multi-layer annotation, allows parallel comparison of two chromatin states, and is compatible with Shiny-based analysis workflows. As multi-omic and spatial genomic datasets grow in complexity, GeomeTriD will facilitate the reconstruction and comparison of 3D genome structures across conditions, linking chromatin architecture to gene regulation, epigenetic states, and cell-state transitions.

**Availability and implementation:**

geomeTriD is freely available at https://bioconductor.org/packages/geomeTriD.

## 1 Introduction

Genomic analysis approaches based on chromosome conformation capture technologies (Dekker *et al.* 2023) are increasingly employed to explore the three-dimensional (3D) organization of genomes and to uncover the regulatory mechanisms of gene expression in spatial and temporal contexts. With the development of single-cell Hi-C maps and the corresponding 3D genome models (Nagano *et al.* 2013, Flyamer *et al.* 2017, Nagano *et al.* 2017, Ramani *et al.* 2017, Stevens *et al.* 2017, Gabriele *et al.* 2022), it has become evident that the 3D genome is highly dynamic within individual nuclei, exhibiting substantial heterogeneity across cells. Understanding the spatial and temporal organization of the genome at the single-cell level is both the current focus and future goal of the 4D Nucleome Project (Dekker *et al.* 2023). Recent advances in single-cell multi-omic approaches (Lee *et al.* 2019, Li *et al.* 2019b, Liu *et al.* 2023, Chai *et al.* 2025) have enabled simultaneous profiling of 3D genome structure, epigenomic states, and transcriptional activity. To fully leverage these complex, multi-layered bulk and single-cell datasets, effective computational tools are essential for integrating, manipulating, and visualizing the spatial and temporal dimensions of genome organization.

Various software tools have been developed to manipulate and visualize chromatin interaction data across different scales, including genome-wide, chromosome-specific, and region-specific levels. At the genome-wide level, interactions are often visualized as heatmaps or link diagrams, as well as aggregate profiles such as Aggregate Peak Analysis (APA), Aggregate Region Analysis (ARA), and Aggregate Topologically Associating Domain Analysis (ATA) (Rao *et al.* 2014). Chromosome-specific views typically include heatmaps, curves, or links aligned to genome coordinates, highlighting features such as compartments and long-range intra-chromosomal interactions. Region-specific visualizations focus on finer structures, such as topologically associating domains (TADs), tethering elements (Batut *et al.* 2022), and chromatin loops, often displayed as 2D heatmaps or interaction arcs, using linear genome browsers [e.g. Juicebox (Durand *et al.* 2016), HiGlass (Kerpedjiev *et al.* 2018), 3D Genome Browser (Wang *et al.* 2018), Nucleome Browser (Zhu *et al.* 2022), and WashU Epigenome Browser (Li *et al.* 2019a)] or circular layouts (e.g. Circos). In the heatmaps, both axes represent genomic coordinates, and color intensity reflects interaction frequency; arc-based representations similarly connect interacting loci with curves color-coded by strength. These visualizations are often integrated with external genomic data formats (e.g. bigWig, bedGraph), aligned to gene models and other annotations. However, 2D contact maps inherently lack the spatial context of the nucleus, limiting the ability to directly interpret chromatin folding, domain separation, and the physical proximity of regulatory elements—challenges that 3D visualization tools are designed to overcome.

The field of 3D chromatin modeling is rapidly advancing, driven by both optimization-based approaches (e.g. manifold learning) and probabilistic methods (e.g. Markov Chain Monte Carlo). Numerous tools are now available to reconstruct 3D genome structures from bulk Hi-C data (Hu *et al.* 2013, Peng *et al.* 2013, Zhang *et al.* 2013, Varoquaux *et al.* 2014, Hirata *et al.* 2016, Szalaj *et al.* 2016, Trieu and Cheng 2016, Serra *et al.* 2017, Abbas *et al.* 2019, Trieu *et al.* 2019, Zhang *et al.* 2019, Li *et al.* 2020, Wang *et al.* 2022, Li *et al.* 2023). Tools like Dip-C, hickit (Tan *et al.* 2018, 2019) and Tensor-FLAMINGO (Wang *et al.* 2025) enable reconstruction of 3D chromosomal architecture at single-cell resolution. Visualization of these models typically relies on JavaScript libraries (e.g. D3.js, three.js, Jmol) or OpenGL. Three.js-based tools like the 3D Genome Browser (3DGB) (Butyaev *et al.* 2015), HiC-3DViewer (Djekidel *et al.* 2017), and g3dtools (Li *et al.* 2022) support interactive navigation of single-omics 3D models. However, existing tools have two main limitations in comparing genomic and omics features across conditions in 3D models (Supplementary Table S1, available as supplementary data at *Bioinformatics Advances* online). First, they lack support for integrated multi-omics visualization within 3D genome structures—critical for understanding links between architecture and regulation. Second, they do not allow side-by-side comparison of multiple 3D models within a unified interface—an essential feature for detecting conserved structures, identifying subtle differences, and interpreting biological processes such as development, regeneration, disease, and environmental responses. As a result, users are forced to open separate sessions, disrupting analytical continuity.

We introduce geomeTriD (Geometry in Three Dimensions), a Bioconductor package designed for interactive visualization of multi-omics data and genomic annotations within 3D genome structures. geomeTriD enhances clarity and interpretability, particularly when comparing genomic features and omics signals across different conditions. The package integrates seamlessly with Shiny, enabling interactive web-based data exploration. Through case studies, we demonstrate that geomeTriD effectively reveals dynamic relationships among genes, regulatory elements, and multi-omics signals in both bulk and single-cell data. By uncovering patterns often missed by other chromatin visualization tools, geomeTriD facilitates more efficient hypothesis generation and refinement.

## 2 Materials and methods

### 2.1 Implementation

The geomeTriD package is developed within the Bioconductor framework (Gentleman *et al.* 2004) using R (Ihaka and Gentleman 1996), and is freely available through multiple channels, including the stable release on the Bioconductor website (https://bioconductor.org/packages/geomeTriD) and the development version. All source code used to generate the figures in this manuscript is available on GitHub (https://github.com/jianhong/geomeTriD_documentation).

To install the geomeTriD package, first download and install R from the official CRAN website (https://cran.r-project.org/doc/manuals/r-release/R-admin.html). After installing R, open an R session and run the following commands.

if (! requireNamespace('BiocManager', quietly = TRUE))

   install.packages('BiocManager')

BiocManager::install('geomeTriD')

Refer to the Bioconductor package page for full dependency details, or seek help through the Bioconductor support site or the GitHub issue page if installation issues occur.

### 2.2 Functionalities of 3D model plots in geomeTriD

The geomeTriD package generates interactive 3D plots using the GL library and the three.js visualization library. Visualizing epigenomic data in 3D with geomeTriD involves three main steps:

1. **Create or load a 3D model** into a GRanges object, including metadata for positions.
2. **Attach multi-omics data** to the 3D model and package them as a list of threeJsGeometry objects.
3. **Visualize the multi-omics data** along the 3D model using the threeJsViewer function powered by three.js.

The mdsPlot function computes spatial positions from a bin-based contact matrix using Kruskal’s Non-metric Multidimensional Scaling (MDS) algorithm. The importGInteractions function from the trackViewer package streamlines the conversion of interaction data from BEDPE, HIC, and COOL formats into a track or GInteractions object, which can then be used as input for mdsPlot. Users are encouraged to explore alternative methods, such as FLAMINGO and hickit, for generating 3D genomic models.

The resulting 3D genomic model can be integrated with multi-omics data—including gene models, coverage signals from BAM, BED, BedGraph, and bigWig files, as well as mutations from VCF files—using the versatile view3dStructure function.

The threeJsViewer function visualizes the 3D model by rendering it as an htmlwidgets widget, with the model packaged in threeJsGeometry objects. This widget supports two layouts:

- **Single Layout**: Displays a single 3D model with top and bottom layers, allowing for the comparison of different signals within the same model.
- **Side-by-Side Layout**: Facilitates the comparison of two 3D models. Prior to visualization, it is recommended to align the coordinates using the alignCoor function.

The widget organizes signals into sub-layers that can be toggled on or off for better visualization. Powered by three.js, the 3D models offer interactive features, allowing users to zoom, rotate, and pan the view seamlessly. Additionally, users can measure the distance between any two annotations within the 3D model for detailed analysis.

### 2.3 Functionalities of cell clustering in geomeTriD

The 3D model clustering operates on two levels: clustering points within each 3D genome model, and clustering entire 3D structures across cells. The pointCluster function applies Density-Based Spatial Clustering of Applications with Noise (DBSCAN) to identify point clusters within a single 3D structure. To cluster 3D structures across cells, the cellCluster function provides three main approaches for computing the distance matrix:

1. Root Mean Square Deviation (RMSD)—calculated after structural alignment to quantify global differences.
2. Cluster similarity metrics—such as Adjusted Rand Index (ARI), Normalized Information Distance (NID), Normalized Mutual Information (NMI), Adjusted Mutual Information (AMI) and Sequence Relabeling Distance (SRD), used to compare DBSCAN-based point clusters between structures. This method is most effective for models with well-separated TAD-like domains.
3. Centroid-based distance—calculated as the Euclidean distance between cells based on the average distance of each point to its cluster centroid. This method is suited for models with known changes in chromatin loops or superloop structures.

Internally, the 3D points are first aligned using the Kabsch–Umeyama algorithm (Kabsch 1976, Umeyama 1991), and then clustered using the dbscan function from the dbscan package(Ester *et al.* 1996). Cluster similarity metrics—such as ARI, NMI, NID and NMI—are computed using functions from the aricode package (Sundqvist *et al.* 2023).

### 2.4 Functionalities of 2D plots for genomic interactions

The loopBouquetPlot function generates a straightforward 2D representation using the layout_with_fr algorithm from the igraph package (Csardi and Nepusz 2006) to determine the layout. In the layout, nodes represent interaction regions and edges represent two types of connections: DNA interactions and gaps between interaction regions. The weight of the edges corresponding to gaps is calculated based on the maximal length of the gaps divided by their respective lengths. DNA interaction edge weights are determined by the interaction score, normalized by the third quartile of gap weights and multiplied by twice the interquartile range. Node size is proportional to the corresponding region length. Dashed outlines highlight DNA interaction hubs formed by densely clustered interaction nodes. Coordinates along the DNA chain indicate the actual fragment lengths.

## 3 Results

The geomeTriD package is designed to visualize chromosome- and region-level chromatin architecture using multiple layouts, supporting direct comparative analyses across conditions with integrated genomic annotations and multi-omics data. To demonstrate its versatility, we present several illustrative examples showcasing the visualization of A/B compartments, TADs, microcompartments, chromatin loops, and *cis*-regulatory elements (cREs), along with associated multi-omics signals at both bulk and single-cell resolution.

### 3.1 Case study 1: visualizing spatial distribution changes of histone markers among A/B compartments

**Figure 1.**
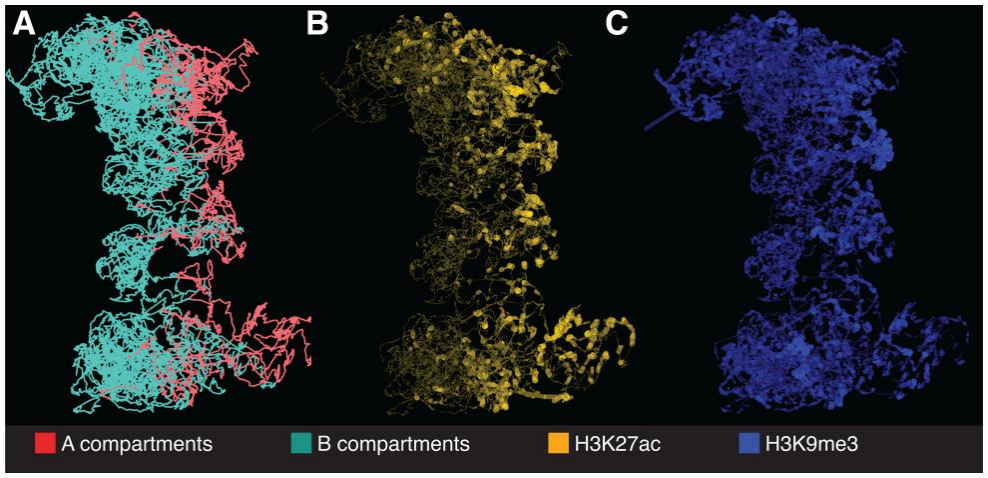
Visualization of A/B compartments and histone modifications in GM12878. A, A/B Compartments. B, H3K27ac distribution. C, H3K9me3 distribution. The color gradient and line width represent signal strength.

On a large scale, chromosomes are partitioned into A (“active”) and B (“inactive”) compartments (Harris *et al.* 2023). In contrast to B compartments, A compartments are gene-rich, GC-rich, and enriched with histone marks linked to active transcription. The geomeTriD package has been used to visualize the distribution of H3K27ac and H3K9me3 along the 3D structures of chromosome 4 in GM12878 cells, generated by FLAMINGO at 5 kb resolution. The 3D plots highlight the correlation of A/B compartments in 3D (Fig. 1). Figure 1A shows that active compartments are positioned on one side of the chromosome, while inactive compartments are on the opposite side, suggesting a dynamic relationship between histone modifications and spatial organization. This spatial organization, where A or B compartments cluster together, cannot be effectively conveyed through 2D heatmaps.

The overlay of histone modification signals, such as H3K27ac (Fig. 1B) and H3K9me3 (Fig. 1C), further validated the model’s capacity to capture key epigenetic features. In particular, the active transcription marker H3K27ac was highly enriched in A compartments, while the repressive histone mark H3K9me3 was more variable but often observed in regions associated with gene silencing, which can include both A and B compartments. This observation aligns with the findings reported by Nichols *et al.* (Nichols and Corces 2021), who suggested that the enrichment of H3K9me3 in A compartments may reflect cell-type-specific features or higher-resolution sub-compartment events associated with H3K9me3. These corroborated findings on the relationship between histone modifications and chromatin compartmentalization emphasized the utility of geomeTriD in visualizing complex genomic features in three dimensions.

We present the distribution of histone markers using different layouts, as shown in the supplemental material: Fig1.part1.html (single layout) and Fig1.part2.html (side-by-side layout), available as supplementary data at *Bioinformatics Advances* online. In the single layout, users can adjust the display ratio between the bottom and upper layers by dragging the slider (indicated by a red horizontal line). In the side-by-side layout, users can compare signals directly by displaying them next to each other. By clicking to open the draggable “Controls” menu, a show/hide menu will appear, allowing users to toggle the visibility of genomic signals by clicking the corresponding buttons. This functionality simplifies the inclusion and comparison of multiple omics data sets. The animation exported from Supplemental Fig1.part1.html, available as supplementary data at *Bioinformatics Advances* online, demonstrates a 360-degree rotation and layer toggling to visualize the corresponding histone marker signals (Supplemental Fig1.part1.exportAsVideo.webm, available as supplementary data at *Bioinformatics Advances* online).

### 3.2 Case study 2: visualizing microcompartments or loops

**Figure 2.**
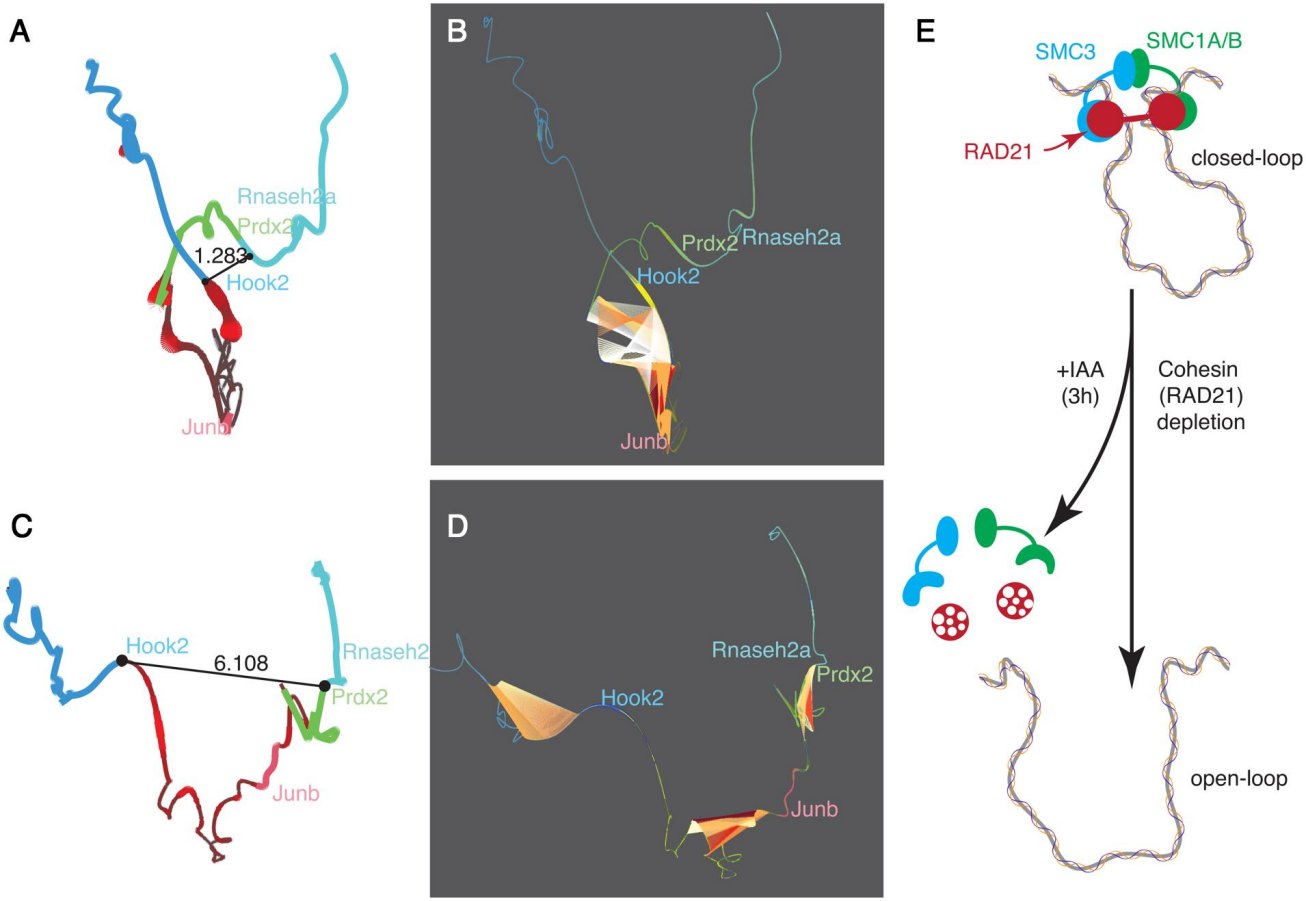
Visualization of microcompartment open and closed states following cohesin depletion. The 3D models display RAD21 ChIP signals (in red) before (A) and after (C) cohesin depletion with the mm39 genome coordinates for chr8:85.69-85.73M. The distance between the *Prdx2* and *Hook2* promoters is annotated as black fragments. The models also highlight the top 10 strongest genomic interactions within the microcompartments, shown as colored fragments linked to interaction sites before (B) and after (D) cohesin depletion. The color gradients, ranging from white to red, indicate the interaction strength. (E) illustrates the treatment paradigm for the rapid depletion of RAD21 following IAA treatment, adapted from Goel *et al.*’s Figure 4.

As reported by Goel *et al.* (Goel *et al.* 2023), a subset of promoter-promoter interactions exhibit either increased or decreased strength following the depletion of RAD21, a subunit of cohesin. In this study, we have applied the geomeTriD package to visualize changes in microcompartments around the *Prdx2* and *Hook2* genes, as observed in Event II of the *Klf1* locus on chromosome 8 in Goel *et al.*’s Fig. 4 (2023). As shown in Fig. 2A and C, the closed loop surrounding the *Prdx2* and *Hook2* genes was disrupted following RAD21 depletion after 3 hours of IAA treatment, increasing the Euclidean distance between promoters from 1.283 to 6.108. While the original study presented this change schematically, our 3D visualization of the predicted structures offers a more direct and intuitive depiction of the loop opening.

In some cases, the 3D structure still appears cluttered, making it challenging to emphasize changes. For example in Supplementary Fig. S3, available as supplementary data at *Bioinformatics Advances* online, it allows us to explore spatial distance variations between a recently identified silencer at genomic coordinates chr5:21.8M (Ando *et al.* 2024) and the *tenm1* gene in samples of zebrafish fins. The loopBouquetPlot function utilizes graph and network analysis to create a clean and visually intuitive representation of data (Supplementary Fig. S3C and D, available as supplementary data at *Bioinformatics Advances* online). Such insights, assessed in freshly injured fins (Supplementary Fig. S3A and C, available as supplementary data at *Bioinformatics Advances* online) and during fin regeneration at 4 days post-amputation (dpa) (Supplementary Fig. S3B and D, available as supplementary data at *Bioinformatics Advances* online), would be difficult to discern in a linear layout (Supplementary Fig. S3E, available as supplementary data at *Bioinformatics Advances* online).

Figure 2B and D and supplemental material Fig2.part2.html, available as supplementary data at *Bioinformatics Advances* online, highlights the top 10 strongest genomic interactions within the microcompartments, represented as colored fragments connected to various genomic locations. Notably, these interactions shift from cohesin-binding regions associated with the closed loop to local, short-distance interactions after RAD21 depletion. This change highlights how cohesin plays a critical role in maintaining the 3D organization of the region.

To demonstrate the generalizability of this approach, we visualized the well-characterized long-range promoter–enhancer interaction between *Sox2* and its distal ∼100 kb enhancer, the *Sox2* Control Region (SCR) (Li *et al.* 2014). As shown in Supplementary Fig. S2, available as supplementary data at *Bioinformatics Advances* online, the close *Sox2*–SCR interaction was disrupted after RAD21 depletion, increasing the Euclidean distance from 2.924 to 9.251. Among the top ten strongest interactions (Supplementary Fig. S2B and D, available as supplementary data at *Bioinformatics Advances* online), the dominant contact shifted from the *Sox2*–SCR pair to other genomic regions, consistent with loop disassembly upon cohesin loss.

Further analysis is presented in the visualization of CTCF, YY1, RAD21, SMC1A, and SMC3 signals along the 3D model of DMSO and IAA (3 h) samples (available in the supplemental material, Fig2.part1.html and Fig2.part2.html, available as supplementary data at *Bioinformatics Advances* online). By toggling the cohesin signals (RAD21, SMC1A, and SMC3), users can dynamically explore the overlapping ChIP signals, while toggling the CTCF and YY1 signals vividly demonstrates their reduced strength upon cohesin depletion. This visualization approach allows for a deeper understanding of the underlying chromatin dynamics.

### 3.3 Case study 3: visualizing single cell 3D models

While bulk Hi-C reveals core principles of 3D genome organization, it averages interactions across cells, masking variability. Single-cell Hi-C overcomes this by capturing chromatin contacts in individual cells, uncovering heterogeneity in TADs and loops across cell types and states. Using single-cell Dip-C data from GM12878 cells (Tan *et al.* 2018) and HiRES data from mouse radial glia cells at the G1 stage (Liu *et al.* 2023), we visualized the maternal and paternal 3D structures of chromosome X (chrX), highlighting previously reported conformational differences that reflect allele-specific chromatin organization.

**Figure 3.**
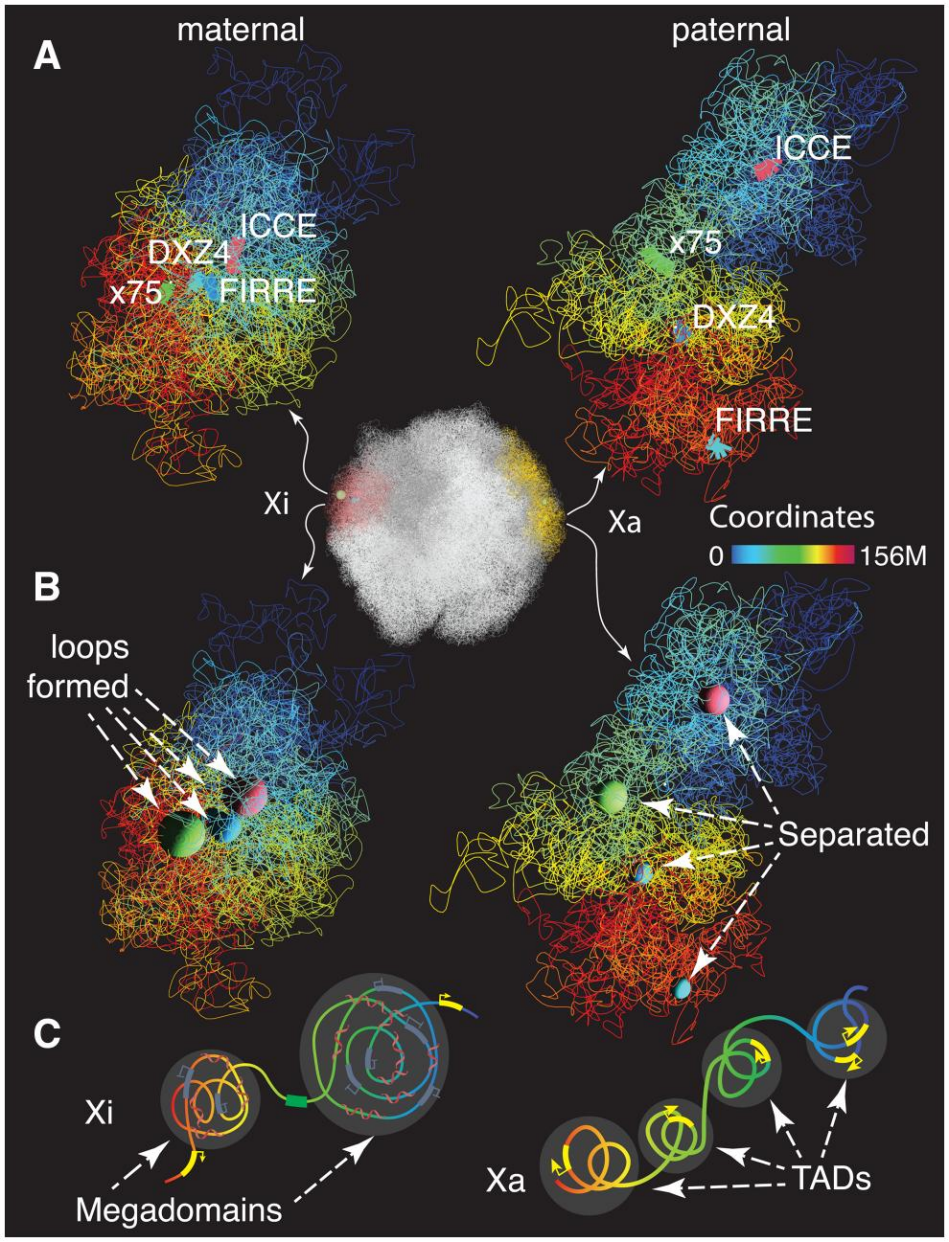
Visualization of active and inactive chrX in human cells at the single-cell level. Left panels show the maternal chrX; right panels show the paternal chrX. A, B: 3D genome structures of GM12878 human cell 3 (Tan *et al.* 2018). A, highlights genomic loci ICCE (hg38: 56.8M), X75 (75.35M), DXZ4 (115M), and FIRRE (130.85M) as linear segments. B, represents these loci as spheres marking superloop anchor points. C, Cartoon illustrations adapted from reference (Loda *et al.* 2022) highlight the canonical structures of Xa and Xi: Xa displays TADs, whereas Xi lacks TADs and instead forms megadomains.

As expected, we reproduced the previously reported finding in female GM12878 cell 3 that the active chrX (Xa) typically exhibits an extended conformation, while the inactive chrX (Xi) appears more compact (Fig. 3). The diploid 3D genome model is shown centrally in Fig. 3. We visualized superloop anchors using two representations: linear segments (Fig. 3A) and spheres (Fig. 3B). The segment lengths or sphere radii reflect the local DNA condensation levels at each superloop anchor. Notably, not all cells show clearly distinguishable Xa and Xi, and superloop formation in Xi is often inconsistent (Supplementary Fig. S4A and B, available as supplementary data at *Bioinformatics Advances* online). Furthermore, allelic configurations vary among cells, with some exhibiting a paternal Xa and others a maternal Xa.

Allelic heterogeneity of Xa and Xi is also observed in mouse cells (Supplementary Fig. S4C and D, available as supplementary data at *Bioinformatics Advances* online) (Liu *et al.* 2023). To illustrate this, we selected two representative cells and visualized both maternal and paternal chrX structures. Leveraging HiRES, which integrates 3D genome structure with gene expression, we overlaid RNA expression data along with genomic annotations—such as the superloop anchors Firre, Dxz4, Xist, and X75—onto the 3D models. RNA expression levels of X-linked genes were mapped onto the allele with the larger spatial extent. Compared to the cell shown in Supplementary Fig. S4C, the cell in Supplementary Fig. S4D, available as supplementary data at *Bioinformatics Advances* online, displays higher expression of X-linked genes and a more relaxed, extended chromatin conformation. To highlight the capabilities of the geomeTriD package to present diverse data types, allele-specific chromatin interactions are shown as dark gray lines.

### 3.4 Case study 4: clustering cells based on 3D models

**Figure 4.**
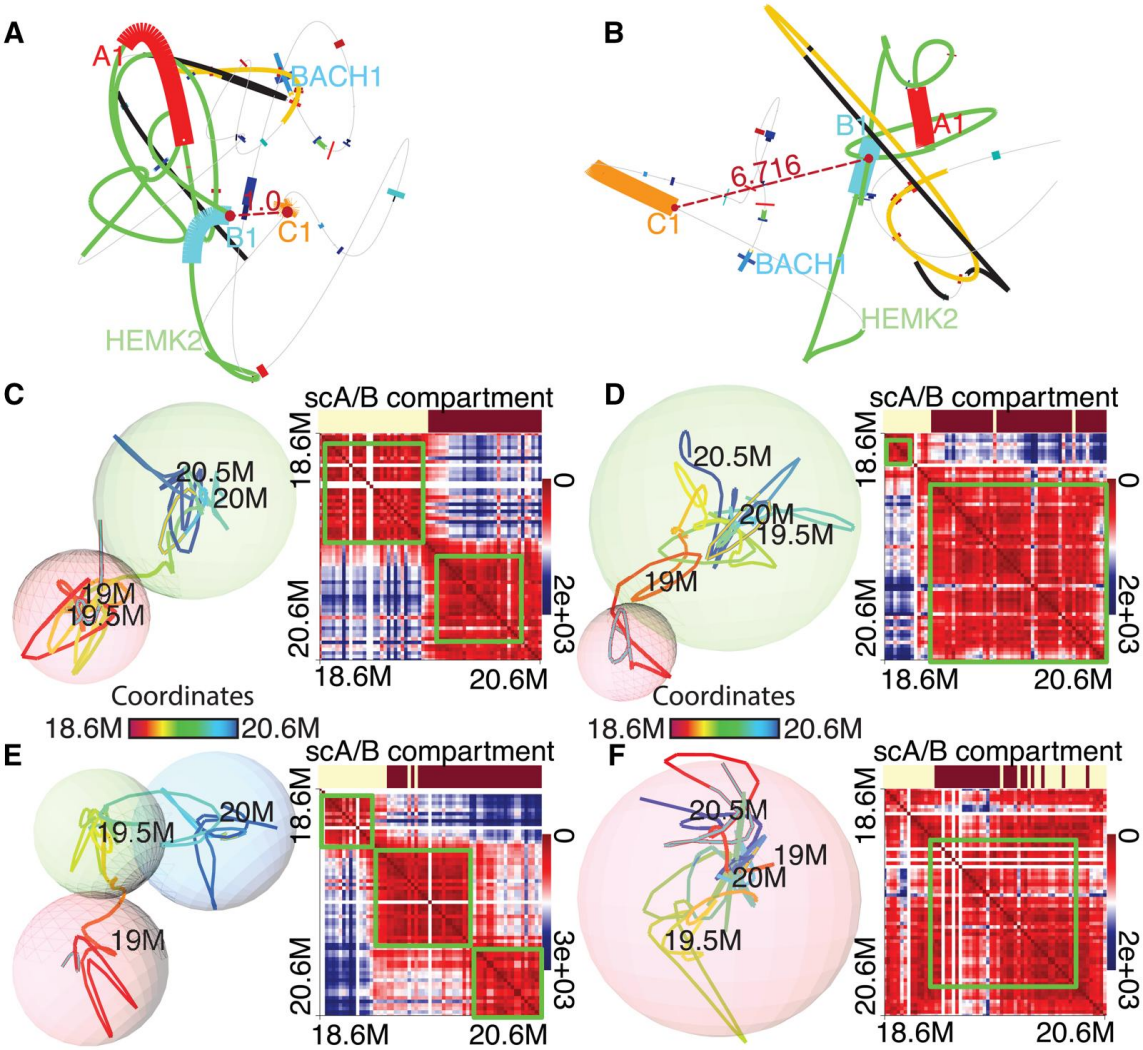
Visualization of clustered 3D genome models for the human IMR90 cell line, focusing on hg19 genomic regions chr21:28.2–30.3 Mb and chr21:18.6–20.6 Mb (Rao *et al.* 2014). A, B: Cooperative interactions among a specific triplet of 30-kb segments starting at positions 28.51M (A1), 28.78M (B1), and 28.93M (C1). Cells are clustered based on the spatial similarity of these three segments, using the Euclidean distance between each cell’s 3D configuration and the centroid structure. Representative 3D models from two of the resulting clusters are shown to highlight differences in the Euclidean distance between B1 and C1. A represents a structure in which A1, B1, and C1 are spatially close, while B shows a representative structure where the three segments are more dispersed. C–F: Single-cell spatial distance matrices (right panels) and corresponding pseudo-colored 3D visualizations (left panels) for four example cells in the STORM-imaged region. The spheres represent point clusters identified via DBSCAN. In the distance matrices, the sc A/B compartments are derived from the first principal component (PC1) of the pairwise Pearson correlation matrix. Green rectangles in the heatmaps indicate clusters matching the DBSCAN-identified point groups shown as spheres on the left.

In single-cell analysis, clustering is a fundamental step for grouping cells with similar molecular profiles, allowing researchers to identify distinct cell types, cellular states, or developmental trajectories from high-dimensional data. In this study, we aim to cluster single cells based on their native 3D chromosomal architecture, as reconstructed from Stochastic Optical Reconstruction Microscopy (STORM) imaging data (Rao *et al.* 2014). We analyzed the 3D chromosomal architecture of the human IMR90 cell line for the region chr21:28.2–30.3 Mb by focusing on three selected 30K segments starting at positions 28.51 Mb (A1), 28.78 Mb (B1), and 28.93 Mb (C1). For each cell, we calculated the sum of the Euclidean distances from A1, B1, and C1 to their centroid, which reflects the spatial compactness of the triplet. Based on these distances, we performed hierarchical clustering to group cells into three categories: small, medium, and large spatial separation among the triplet. To represent each cluster, we selected the cell with the smallest average distance to all other cells in the same group. Representative 3D models from the clusters with the smallest and largest distances were visualized. In Fig. 4A, the Euclidean distance between B1 and C1 is 1.0, whereas in Fig. 4B, it increases to 6.716, demonstrating the effectiveness of the cell clustering.

In the previous case, we focused on a specific genomic region of interest, but often such prior knowledge is unavailable. To cluster cells unsupervised based on their 3D chromatin structures, the geomeTriD package offers several distance metrics beyond centroid-based distance, including RMSD and cluster similarity metrics. Here, we applied the NID metric to DBSCAN-derived point clusters for the human IMR90 cell line at chr21:18.6–20.6 Mb. We identified several cell subsets containing two or more TAD- or subTAD-like structures and present four representative cells exhibiting distinct TAD/subTAD-like architectures (Fig. 4C–F). The point clusters identified by DBSCAN correspond well with the first principal component (PC1, shown as the scA/B compartment in the right panels) derived from the pairwise Pearson correlation matrix of the spatial distance matrix. When combined with 3D visualization, gene annotations, chromatin accessibility, and epigenetic data, these point clusters offer insight into gene regulation within TAD- or subTAD-like structures.

In Supplementary Fig. S5A–C, available as supplementary data at *Bioinformatics Advances* online, we show ATAC-seq and RNA-seq signals mapped onto allele-specific 3D models of chrX: 1–20 Mb in Patski cells (a hybrid mouse fibroblast cell line) across cell cycle pseudotime: G1, S, and G2/M (Chai *et al.* 2025). Notably, some point clusters contain multiple highly expressed genes, often accompanied by open chromatin regions upstream of the gene loci. Additionally, we observed a reduction in the physical size of the chrX in G2/M compared to G1 and S phases, consistent with progressive chromatin condensation during the cell cycle.

## 4 Discussion

The geomeTriD package provides interactive ways to visualize chromatin interactions and gene regulation, allowing researchers to investigate spatial dynamics that are often obscured in traditional 2D plots. In this study, we demonstrate how these tools can uncover changes in spatial distances and interactions across various chromatin contexts at both bulk and single-cell levels.

Our package supports visualization of either predefined TADs (Fig. 3) or TAD-like structures automatically inferred from reconstructed 3D chromatin models (Fig. 4). Similarly, users can visualize predefined compartments (Fig. 1) or those inferred *de novo* via PCA. For unsupervised detection of TAD-like structures, the package incorporates DBSCAN clustering to identify spatial point clusters, which are rendered as segments or transparent spheres within the 3D genome model. Both representations highlight that TADs are spatially extended, not point-like. By revealing their size, shape, and position, our methods uncover key biological patterns—such as overlaps of regulatory elements and boundaries, gene co-localization, and spatial separation of functional compartments crucial for long-distance interactions.

To evaluate the accuracy of a predicted 3D genome model for a specific region, a visual comparison between the spatial distance matrix (derived from the model) and the corresponding Hi-C contact map can be used as an intuitive alternative to numerical metrics like reconstruction error or distance correlation. This side-by-side visualization reveals how well the model captures topological features such as domains, loops, and long-range interactions. High contact frequencies in the Hi-C map align with low spatial distances in the model, indicating regions of close physical proximity. As shown in Supplementary Fig. S6A, available as supplementary data at *Bioinformatics Advances* online, the Hi-C map captures interaction frequencies across chrX: 1–20 Mb, while Supplementary Fig. S6B, available as supplementary data at *Bioinformatics Advances* online, shows the corresponding spatial distance matrix computed from FLAMINGO-generated 3D coordinates. This geometry-aware representation provides a complementary and user-friendly way to validate predicted structures without relying solely on statistical measures, enabling researchers to quickly assess model quality and detect potential discrepancies in specific subregions.

Reconstructing 3D chromosomal architecture at single-cell resolution using imputation techniques addresses the challenge of data sparsity, enhances structural resolution, preserves cell-to-cell variability, and enables integrative downstream analyses that link chromatin organization to gene expression, epigenetic states, and cellular phenotypes. These improved 3D models facilitate clearer visualization, more accurate comparisons across cells or conditions, and the detection of structural patterns such as allele-specific folding and transitions during development. In this study, we visualized both active and inactive X chromosomes in human and mouse cells at the single-cell level (Fig. 3, Supplementary Fig. S4, available as supplementary data at *Bioinformatics Advances* online), demonstrating how the integration of 3D genome modeling with multi-modal genomic data holds significant potential for uncovering the spatial basis of gene regulation and cellular heterogeneity.

To cluster single-cell 3D genome conformations, we applied various distance-based approaches. When specific genomic regions of interest—such as known interacting loci—are available, centroid-based distances can effectively quantify spatial proximity and group cells accordingly (Fig. 4A and B). When prior knowledge is lacking, the geomeTriD package offers alternative metrics—including RMSD and several cluster similarity measures-such as ARI, NID, NMI, AMI, and SRD—to enable unsupervised clustering based on global 3D chromatin architecture (Fig. 4C–F). The cellCluster function also supports the use of cluster similarity metrics on single-cell chromatin A/B compartment scores (scA/B, represented by PC1 in geomeTriD) across large genomic regions. A detailed comparison of these clustering methods is not included in this manuscript, as it falls outside the scope of the current study.

Clustering not only uncovers previously uncharacterized cell populations but also provides a foundation for downstream analyses by enabling signal aggregation within each group. However, when applied to single-cell 3D genome models, aggregating spatial coordinates can be highly sensitive to outliers, even after proper structural alignment. Furthermore, if the 3D conformations are computationally predicted, the reliability of such aggregation is further constrained by the accuracy of the underlying models. To address these challenges, we visualize representative cells from each cluster in this study, rather than aggregating all structures, to more faithfully reflect the diversity and integrity of the 3D chromatin architectures.

In conclusion, the geomeTriD package provides a powerful and flexible platform for visualizing multi-omic data within the context of 3D genomic structures. Unlike traditional approaches that focus on a single type of omics data, geomeTriD supports the integration of diverse datasets, including gene annotations, chromatin accessibility, gene expression, and other epigenetic signals. The package also implements multiple distance-based clustering methods, enabling users to identify distinct chromatin states based on 3D structural similarity. Through its interactive HTML widget, users can easily toggle between different data layers to display or hide specific omics signals, enhancing the interpretability of complex spatial relationships. The tool also supports precise measurement of spatial distances between any two genomic annotations, providing valuable insights into the proximity and potential interactions of regulatory elements and target genes. Furthermore, the HTML widget is fully compatible with Shiny applications, offering a user-friendly and interactive interface for exploring single-cell 3D genome models. This combination of integrative visualization, interactive exploration, and detailed spatial analysis makes geomeTriD a valuable resource for researchers aiming to uncover the spatial and functional organization of the genome across diverse biological conditions.

## Supplementary Material

vbaf299_Supplementary_Data

## Data Availability

Dataset for case study 1: The data were downloaded from ENCODE for GM12878 with assembly hg19. The 3D structure model was downloaded from FLAMINGO results for Gene Expression Omnibus dataset (GEO) dataset with accession GSE63525. Dataset for case study 2: The Region Capture Micro-C data were downloaded from GEO (GSE207225). The genomic signals of ChIP-seq were downloaded from GEO (GSE178982) and remapped to mm39 genome by nfcore/chipseq pipeline (https://nf-co.re/chipseq/2.0.0/). Dataset for case study 3 and Supplementary Fig. S4: The human Dip-C data were downloaded from GEO (GSE117874). The HiRES data were downloaded from GEO (GSE223917). Dataset for case study 4 and Supplementary Fig. S5: The STORM data for IMR90 were download from github (BogdanBintu/ChromatinImaging). The Tri-omic data were downloaded from Genome Sequence Archive in National Genomics Data Center with accession number PRJCA024774 and analyzed by Dip-C commit ea516eb. Dataset for Supplementary Fig. S3: HICAR data were downloaded from GEO (GSE231771) and analyzed by nfcore/hicar pipeline (https://github.com/jianhong/hicar/releases/tag/2.0.0rc).

## Code availability

geomeTriD is freely available at https://bioconductor.org/packages/geomeTriD and GitHub (https://github.com/jianhong/geomeTriD). The scripts to generate the plots are available at https://github.com/jianhong/geomeTriD_documentation.
