# Supplementary material for: geomeTriD: a Bioconductor package for interactive and integrative visualization of 3D structural model with multi-omics data
Source: Bioinform Adv. 2025 Nov 23;5(1):vbaf299. doi: 10.1093/bioadv/vbaf299 (PMC12702139; doi:10.1093/bioadv/vbaf299)
Supplement: vbaf299_Supplementary_Data [file vbaf299_supplementary_data.zip › Sup.Fig1.part2.html.zip!/Sup.Fig1.part2.html]

threeJsViewer
